# Acupoint catgut embedding for patients with chronic urticaria: A systematic review protocol

**DOI:** 10.1097/MD.0000000000016036

**Published:** 2019-06-14

**Authors:** Yunzhou Shi, Tinghui Hou, Qianhua Zheng, Ying Liu, Ting Yang, Ying Li

**Affiliations:** Department of Acupuncture and Moxibustion, Chengdu University of Traditional Chinese Medicine, Jinniu District, Chengdu, Sichuan, China.

**Keywords:** acupoint catgut embedding, chronic urticaria, protocol, systematic review

## Abstract

**Background:**

The purpose of this paper is to evaluate the effectiveness and safety of acupoint catgut embedding in the treatment of chronic urticaria (CU).

**Methods:**

We will electronically search PubMed, Medline, Embase, Web of Science, the Cochrane Central Register of Controlled Trials, China National Knowledge Infrastructure, Chinese Biomedical Literature Database, Chinese Scientific Journal Database, and Wan-Fang Database from their inception to March 2019. In addition, we will manually retrieve other resources including the reference lists of identified publications, conference articles, and gray literature. The clinical randomized controlled trials or quasi-randomized controlled trials related to acupoint catgut embedding for the treatment of CU will be included in the study. The language is limited to Chinese and English. Research selection, data extraction, and research quality assessment will be independently completed by 2 researchers. Data were synthesized by using a fixed effect model or random effect model depend on the heterogeneity test. The total effective rate was the primary outcomes. Skin disease quality of life index scores, adverse events, and recurrence rates will also be assessed as secondary outcomes. RevMan V.5.3 statistical software will be used for meta-analysis. If it is not appropriate for a meta-analysis, then a descriptive analysis will be conducted. Data synthesis will use the risk ratio and the standardized or weighted average difference of continuous data to represent the results.

**Results:**

This study will provide a high-quality synthesis to assess the effectiveness and safety of acupoint catgut embedding for patients with CU.

**Conclusion:**

This systematic review will provide evidence to judge whether acupoint catgut embedding is an effective intervention for patients with CU.

**Systematic review registration:**

PROSPERO, CRD42019129459.

## 1 Introduction

Urticaria is a condition characterized by the development of wheals (hives), angioedema, or both.^[1]^ Physiopathology of urticaria has not been completely elucidated, but the role of histamine, as well as several other mediators, such as platelet-activating factor and cytokines released by activated mast cells and basophils in wheal formation, is undisputed. Chronic urticaria (CU) is defined when an individual presents with transient wheals lasting more than 6 weeks in duration almost daily.^[2,3]^ This is further classified into 2 subgroups: chronic spontaneous urticaria (CSU), which appears not to be triggered by any recognizable external factor and chronic inducible urticaria, triggered by external specific factors, such as a mechanical stimulus (friction, pressure, and vibration), thermal stimulus (cold, heat), aquagenic stimulus (water), and electromagnetic stimulus (solar radiation).^[4]^ The guidelines recommend for only limited extended diagnostic measures in CSU based on patient history.^[1]^ And the important diagnostic step of CU includes a thorough history, physical examination and a ruling out of severe systemic disease. According to recent studies, CSU can occur at any age. The proportion of women in men is 2:1, and the prevalence rate is between 0.5% and 1%.^[5]^ The disease has an impacting heavily on patients’ quality of life and interpersonal relationships.^[6–9]^ Evidence suggested that patients with CSU can suffer from a considerable loss of productivity at work, school, or daily activities.^[10–12]^ There are high direct and indirect health care costs for treating CU due to the large socioeconomic implications of a 20% to 30% reduction in performance.^[13]^

The current treatment guidelines^[1,14]^ and consensus statement^[3,15]^ recommend a stepwise approach for the complete control of CU symptoms. The European Academy of Allergology and Clinical Immunology, the Global Allergy and Asthma European Network, World Allergy Organization (EAACI/GA2LEN/EDF/WAO) guidelines recommend the use of second-generation H1-antihistamines as the first line of treatment. If there is no response at a regular dose, the dose will be increased up to a 4-fold standard or licensed dose. If the response is still no improvement, the guidelines recommend the use of omalizumab and cyclosporine A (CsA) as the third-line treatment. However, a narrative medicine project in Italy showed that the therapeutic pathways were described as unsatisfactory in 83% of the included cases.^[16]^ Many patients do not respond adequately to most of these drugs.^[17]^ Furthermore, the guidelines do not guide the choice, dose, and duration of alternative treatment options in patients who still remain symptomatic despite the use of H1-antihistamines. Also, although the omalizumab and CsA proved to be effective,^[18–20]^ the prices are expensive and can impose a serious economic burden on patients. Widespread use will depend on legal and economic factors.^[21]^ Therefore, an increasing number of patients have sought nonpharmacological treatments.

Acupuncture is an important part of Complementary and alternative medicine, which has a history of more than 2000 years in China. Acupuncture therapy is widely used in the treatment of urticaria due to confirmed efficacy and few adverse effects.^[22]^ And acupoint catgut embedding is a kind of modern acupuncture. It refers to the use of sterile tweezers to put 3-0 catgut (1–1.5 cm) into the needle tip of No. 9 disposable sterile needles, the catgut is parallel with the inner edge of the needle tip, and the needle is followed by a blunt acupuncture needle, inserting the sterile needle into the disinfected acupuncture points. After getting de-qi, the acupuncture needle is pushed in a while withdrawing the sterile needle, leaving the catgut at the acupuncture point.^[23,24]^

Acupoint catgut embedding combines the advantages of acupuncture, acupoint, and thread. It can induce an allergic reaction of the human body, sensitize lymphoid tissue, produce a comprehensive and lasting effect on acupoints and organism, accelerate blood circulation and lymphatic reflux, enhance local tissue activity and metabolism.^[25–27]^ Because its effect can last as long as 20 days and make up for the shortcomings of a long treatment cycle, short stimulation time of acupuncture therapy. Besides, in terms of reducing treatment cost and time, acupoint catgut embedding is better than traditional acupuncture.^[28]^ Nowadays, there have been more and more studies on acupoint catgut embedding in the treatment of CU. However, so far, as we all know, there is no systematic review (SR) at home and abroad to evaluate the efficacy and safety of acupoint catgut embedding in the treatment of CU. Therefore, according to a rigorous review method, we intend to perform a SR to evaluate the effectiveness and safety of acupoint catgut embedding for patients with CU. We hope that we could provide a convincing conclusion.

## 2 Methods and analysis

Our SR is designed in strict compliance with the preferred reporting items for systematic reviews and meta-analysis protocol (PRISMA-P).^[29]^ The PRISMA guidelines and the Cochrane Handbook will be used for us to evaluate the included studies. Besides, our SR will carry out bias risk analysis, heterogeneity analysis. If necessary, subgroup analysis and sensitivity analysis will be conducted. The protocol for this SR has been registered on PROSPERO with registration number: CRD42019129459.

### 2.1 Inclusion criteria

#### 2.1.1 Types of participants

Patients with diagnosed CU will be included, regardless of gender, age, race, education status, and cases of the source. All participants included in the SR must comply with the EAACI/GA2LEN/EDF/WAO guidelines^[1]^ or the Chinese Guidelines about the diagnosis and treatment of urticaria.^[30,31]^

#### 2.1.2 Types of interventions

Intervention measures should be either acupoint catgut embedding alone or combined with other methods to treat CU. If combined with other methods, only the control group with the same intervention measures as the experimental group will be included.

#### 2.1.3 Types of studies

Randomized controlled clinical trials and quasi-randomized controlled trials will be included. We will exclude any other types of literature including literature on acupoint catgut embedding as non-major interventions, retrospective research literature, repeated publication literature, conference abstracts, literature that data cannot be extracted, case reports, and bibliometrics research. Owing to the language restriction of our researchers, we will limit the language of search literature to Chinese and English.

#### 2.1.4 Types of outcomes

The primary outcomes will be the total effective rate. According to the severity of clinical symptoms using 4 scores; the total score is the sum of the individual scores. Symptom score reducing index = (total score before treatment − total score after treatment)/total score before treatment × 100%

The secondary outcomes will include the following measures:

(1) Skin disease quality of life index score
(2) Recurrence rate
(3) Adverse events

### 2.2 Data sources and search methods

#### 2.2.1 Electronic searches

This study will use computer search Medline, Embase, Pubmed, Web of science and the Cochrane Central Register of Controlled Trials. In addition, we will also collect 4 databases of China: China National Knowledge Infrastructure, China Biomedical Literature Database, China Science Journal Database, and Wan-fang Database. All databases will be searched from the date of creation to March 2019. The following search terms will be used: Urticaria, Chronic urticaria, Hives, Nettle-rash, Angioedema, Rubella, Wind cluster, Catgut embedding at acupoint, Catgut implantation at acupoint, Catgut embedding, Acupoint catgut embedding, Catgut. The example search strategy in Table 1 will be used for Pubmed. This search strategy will be slightly modified and used in several other databases.

**Table 1:**
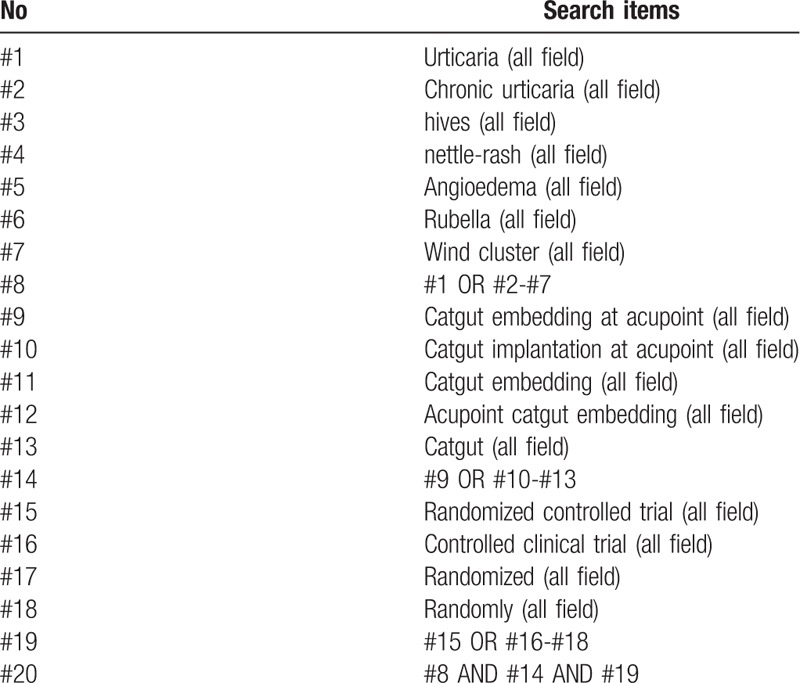
Search strategy used in PubMed.

#### 2.2.2 Searching other resources

We will search the list of the related references for additional trials. The PubMed and Cochrane Library will be searched for existing SRs related to our topic to search their reference lists for more studies. We will also search a reference list for identifying published journals, books, conference articles and gray literature related to this research topic.

### 2.3 Data collection and export

**Figure 1:**
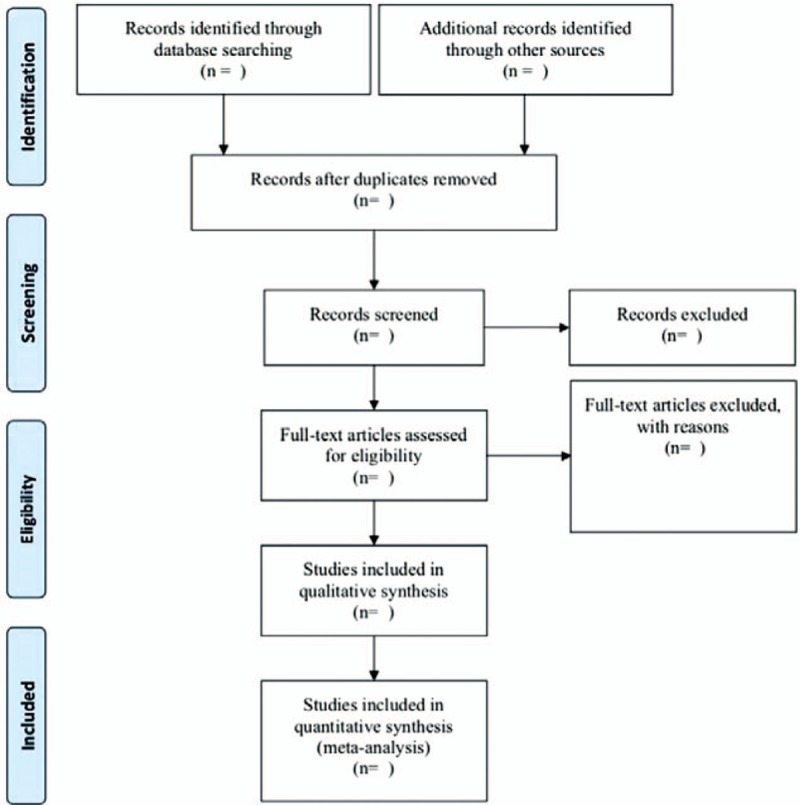
Quorum flow diagram for study retrieval and selection.

After completing all the search work, the results will be exported to Noteexpress software Version 2.6.1 (Aegean Sea software company Beijing, China), and repetitive studies will be deleted by the software. The process of filtering documents will be completed independently by 2 reviewers (LY and YT) and then cross-checked to determine the final inclusion of the documents. In the first stage, all the documents in the search results will be screened for titles, abstracts, and keywords to determine which tests meet the selection criteria. In the second stage, we will evaluate the full text of the study and determine whether it is eligible for SRs. Studies excluded after reading the full text will also be documented and explained why they were excluded. When differences arise at any stage, we will invite the third reviewer to discuss arbitration. The research flow chart is shown in Figure 1.

### 2.4 Data extraction and analysis

Data extraction and analysis will be done by 2 researchers (LY and YT) independently, and the results will be cross-matched. When the differences and opinions are inconsistent, they should be settled through discussion. If the differences encountered cannot be resolved through discussion, a third author (ZQH) will be invited to resolve them. We will make an Excel to extract literature data, which includes the first author, country, year of publication, patient characteristics, number of participant, interventions, outcome, results, main conclusions, conflicts of interest, ethical approval, and other information. If the reported data are not sufficient, we will contact the author of the experiment for consultation and solution.

### 2.5 Assessment of risk of bias in the included studies

We will use the Risk of bias tool in Cochrane Manual V.5.1.0 to evaluate the bias risk of each included studies. The contents include: random sequence generation, allocation sequence concealment, blinding of participants and personnel and outcome assessors, incomplete outcome data, selective outcome reporting, and other sources of bias. The assessment results will be divided into 3 levels: low risk, high risk, and uncertain risk.

### 2.6 Assessment of heterogeneity

The heterogeneity of data will be tested by calculating the value of the *I*^2^ statistic. The study is not considered to have large heterogeneous when the *I*^2^ value is less than 50%. However, when the *I*^2^ value exceeds 50%, there is significant statistical heterogeneity among the trials, and meta-analysis will not be performed. At this time, subgroup stratification analysis is needed to explore the possible causes of heterogeneity.

### 2.7 Assessment of reporting biases

We will use funnel charts to assess reporting biases. When a sufficient number of included studies (at least 10 trials) are available, we will conduct a test for funnel plot asymmetry using the Egger method.^[32]^

### 2.8 Data synthesis

The data synthesis will be performed by using the RevMan V.5.3.The results will be expressed as risk ratio and the standardized or weighted average difference of continuous data. The specific methods are as follows: If the *I*^2^ test is less than 50%, the fixed-effects model will be used for data synthesis. If the *I*^2^ test is between 50% and 75%, the random-effects model will be conducted for data synthesis. If the *I*^2^ test is higher than 75%, we will investigate possible reasons from both clinical and methodological perspectives to conduct subgroup analysis. If data cannot be synthesized, we will provide a descriptive analysis to solve this problem.

### 2.9 Subgroup analysis

In the case of high heterogeneity, we will conduct a subgroup analysis to identify the sources of heterogeneity. Besides, according to different combinations of acupoint catgut embedding, different course time, subgroups of CU, or other factors affecting the results, we will also make subgroup analysis.

### 2.10 Sensitivity analysis

In order to test the robustness of the main decisions in the review process, we will conduct a sensitivity analysis. The main analysis points include the impact of method quality, sample size, and missing data on the study. The meta-analysis will be reused, and more inferior quality studies will be excluded. The results will be compared and discussed according to the results.

### 2.11 Grading the quality of evidence

The quality of SRs will be evaluated by using the grading of recommendations assessment, development, and evaluation.^[33,34]^ Five downgrading factors including risk of bias, inconsistency, indirectness, imprecision, and publication bias will be assessed. The assessment results will be divided into 4 levels: high, moderate, low, or very low.

## 3 Discussion

CU is a complex disease with different pathological mechanisms and inducing factors. Many patients and some dermatologists are unsatisfactory for the therapeutic pathways.^[16,35]^ With the development of complementary and alternative medicine, acupoint catgut embedding, as a product of the combination of traditional acupuncture and modern medicine, has the characteristics of simple operation, long curative effect, low cost, no obvious side effects. And it is widely used in the treatment of CU. Nevertheless, currently, there isn’t a SR-related to acupoint catgut embedding for CU has been published in English. This study will collect evidence comprehensively, extract and analyze the data, and then draw reasonable conclusions, hoping to provide convincing evidence for patients and clinicians during the decision-making process.

## Author contributions

SYZ and HTH contributed to the conception of the study. SYZ drafted the manuscript, and HTH revised the manuscript. The search strategy was developed by all the authors and will be performed by YT and LY, who will also independently screen the potential studies, extract data from the included studies, assess the risk of bias and complete the data synthesis. ZQH will arbitrate in cases of disagreement and ensure the absence of errors. All authors approved the publication of the protocol.

**Conceptualization:** Yunzhou Shi.

**Data curation:** Ying Liu, Ting Yang.

**Formal analysis:** Qianhua Zheng.

**Funding acquisition:** Ying Li.

**Investigation:** Tinghui Hou.

**Methodology:** Yunzhou Shi.

**Project administration:** Ying Li.

**Supervision:** Qianhua Zheng.

**Writing – original draft:** Yunzhou Shi.

**Writing – review and editing:** Tinghui Hou.
